# Climate change and coastal mega cities of India

**DOI:** 10.4103/0019-5278.64612

**Published:** 2010

**Authors:** Harshal T Pandve

**Affiliations:** Department of Community Medicine, Smt. Kashibai Navale Medical College, Pune, India

Sir,

Climate change is one of the most critical global challenges of our times. Recent events have emphatically demonstrated our growing vulnerability to climate change.[1] The United Nation's Intergovernmental Panel on Climate Change (IPCC) 2007 report had projected that sea levels could rise by 18 to 59 cm by 2099. Subsequent studies of glacial melts in Greenland and Antarctica had raised fears that rise in sea levels could be much higher than that indicated in IPCC's 2007 report. Since 1870, global sea level has risen by about 20 cm at an average rate of 1.7 mm/year. But in recent decades, the rate has risen sharply to 2.5 mm/year, according to the latest figures. The rise in sea level is mainly a result of thermal expansion of the ocean due to global warming, as well as increased water inflows from melting glaciers and ice caps.[2] As a result, climate change is fast turning out to be a big challenge for mega cities. Many of the world's largest and fastest-growing cities are located on the coast and therefore vulnerable to sea-level rise. Coastal mega cities are exposed to the more frequent severe windstorms; the heavy rains often result in intense, and sometimes lethal, flash floods. Many waterborne and vector-borne infectious diseases are strongly influenced by climatic conditions, and several are common within cities. More speculatively, global environmental changes may favor the emergence of new infectious diseases, which may spread faster within and between cities due to travel links and higher rates of person-to-person contact.[3]

India has a large coastline. Length of the coastline of India including the coastlines of Andaman and Nicobar Islands in the Bay of Bengal and Laksha dweep Islands in the Arabian Sea is 7517 km. Length of coastline of Indian mainland is 6100 km. Coastline of Indian mainland is surrounded by Arabian Sea in the west, Bay of Bengal in the east and Indian Ocean in the south.[4] The mega cities like Mumbai, Chennai are especially prone to bear the brunt of climate change. A study entitled "Climate change and its economic impact on Mumbai" conducted by the Mumbai office of National Environmental Engineering Research Institute (NEERI) commented that Mumbai, the financial capital of India, could face damages worth Rs. 35,00,000 crores by 2050 because of climate change. Between 1901 and 2007, it registered a mean temperature rise of 1.62°C. The sea level around the island city is rising by 2.4 mm every year. Together they would unleash a chain of disasters such as flash floods, disease outbreaks, building collapses, dislocation and death. High temperatures and a moisture-laden atmosphere would lead to high humidity, increasing the prevalence of vector-borne diseases. By 2050, lung diseases like asthma and various allergies, associated with rapid growth of fungi like Aspergillus and Alternaria, would be common. There would be a cumulative loss of income due to a surge in diseases like malaria, diarrhea and leptospirosis. Increase in the incidence of malaria, diarrhea and leptospirosis would result in loss of income due to nonworking days and deaths. Losses have been computed using disability-adjusted life years (DALYs) for all the major illnesses likely to impact the population. Incidence of all these illnesses will increase steadily with increase in income loss; a sharp increase is likely from 2045 to 2055. By 2050 the cumulative income loss due to malaria, diarrhea and leptospirosis, calculated on the basis of DALYs, would be 155, 597 and 2401 crores, respectively. The calculation of DALYs is based on the World Health Organization (WHO) guidelines and income levels prevalent in Mumbai.[5]

A recently released World Wide Fund (WWF) for Nature report titled "Mega-Stress for Mega-Cities: A Climate Vulnerability Ranking of Major Coastal Cities in Asia" focuses on 11 key Asian cities most likely to be affected by climate change. These cities are Dhaka (Bangladesh), Jakarta (Indonesia), Manila (Philippines), Kolkata (India), Phnom Penh (Cambodia), Ho Chi Minh city (Vietnam), Shanghai (China), Bangkok (Thailand), Hong Kong (China), Kuala Lumpur (Malaysia), and Singapore. Kolkata has a high overall vulnerability score of 7/10. On environmental exposure, it scores 6, with the threat from sea level rise being a dangerous 8 and flooding/drought being 7. Its ability to adapt is also rated as low. Being situated on the banks of the Hooghly River, and within the Ganges delta, Kolkata is vulnerable because it is only meters above current sea levels. As it expanded, it reclaimed significant amounts of surrounding wetland. Consequently, the city sits on alluvial deposits and is within a considerable seismic zone. It is thus prone to earthquakes. As per the report, Kolkata has seen an increase in temperature of around 0.68°C over the last century. Annual mean temperatures are higher after the monsoon and during winter. Increases in sea level accompanied by ground subsidence are the biggest threat to the city, says the report. In fact, a 1-meter rise in sea level could potentially inundate 5763 km in India. In addition to sea level rise, a ground subsidence of 0.6 to 1.9 mm per year is adding to the risk in the Ganges delta. Due to the combined effects of sea level rise and subsidence, the Ganges delta will likely see saltwater intrude 100 km from the coast, greatly impacting ground water supplies. Compounding the effects of saltwater intrusion, overexploitation of ground water in and around Kolkata has led to a drop in its level, leading to further intrusion of seawater, thus making the subsurface ground water saline. Droughts have been more frequent in the last few decades and are projected to get worse, which will lead to even more saltwater intrusion and thus deteriorate surface water and ground water quality. In fact, India may reach a state of severe water shortage and stress before 2025, when the water availability per capita is projected to drop below 1000 meters per year, compared to the level of 1,820 meters per year in 2001.[6]

To conclude, the urban populations in developing countries are vulnerable to climatic threats to health . Specific health vulnerabilities range from heat waves and air-pollution impacts to sea-level rise and storms in coastal cities and to emerging infectious diseases. Climate change has brought additional attention to urban planning and technological choices in the energy and transport sectors, providing an opportunity for greater engagement by the health sector. Planners in developing countries also have the benefit of an emerging understanding of how alternative systems in developed countries have impacted health and the environment. Advances in assessment methods are needed, however, to make better linkages between environmental considerations and urban health and health equity.[3] It is essential for the health policy planners and administrators to consider climate change as a major public health problem in the near future.[7] Health and ecological sustainability would also be enhanced by more active promotion of the "healthy cities" concept, supporting not just an absence of disease but a physical and social environment that enhances all aspects of physical and mental well-being.[3] The inter-sector coordination is the key in dealing with climate change.
